# Enhanced graph attention network by integrating Long Short-Term Memory for artificial emotion representation in multi-modality datasets

**DOI:** 10.1371/journal.pone.0339946

**Published:** 2026-04-27

**Authors:** Weiguang Dong, Mingxin Dong, Yucheng Zhao, Shen Pan

**Affiliations:** 1 Department of Transportation, School of Transportation and Vehicle Engineering, ‌‌Wuxi University, Wuxi, China; 2 Department of Public Security, Shandong Police College, Jinan, China; 3 Department of Research and Development, Jiangyin ZeroPoint Interconnect Technology Co., ‌‌Ltd., Wuxi, China; PLOS: Public Library of Science, UNITED KINGDOM OF GREAT BRITAIN AND NORTHERN IRELAND

## Abstract

Emotion representation is a critical aspect of artificial intelligence, particularly in human-computer interaction and affective computing. Emotion recognition from multi-modal data remains challenging due to the complex semantic relationships between textual, audio, and visual features. This study proposes a hybrid model combining Enhanced Graph Attention Networks and Bidirectional Long Short-Term Memory to address this challenge. First, E-GAT captures structural dependencies between emotional features by constructing a semantic graph from text embeddings. Second, Bi-LSTM models temporal dynamics of sequential data, enabling effective integration of contextual information. We evaluated the model on three benchmark datasets: SemEval-2018 (text-only), RAVDESS (audio-visual), and CMU-MOSEI (multi-modal). Experimental results show that the proposed model achieves state-of-the-art performance: 58.5% accuracy and 68.7% F1-score on SemEval-2018, outperforming baseline models. On multi-modal datasets, it achieves 78.9% accuracy (RAVDESS) and 82.3% accuracy (CMU-MOSEI), demonstrating robust cross-modal generalization. This work advances emotion recognition by providing a unified framework for both text-only and multi-modal scenarios, with applications in human-computer interaction and mental health monitoring.

## 1. Introduction

Emotional intelligence (EI) enables AI systems to perceive, understand, and respond to human emotions, driving applications in human-computer interaction, virtual assistants, and mental health monitoring [1]. However, accurate emotion representation remains challenging due to two key factors: The dynamic, context-dependent nature of human emotions [2]; The complex semantic relationships between multi-modal features (text, audio, vision) [3].

Numerous models have been presented for the portrayal of emotions [4,5]. In general, these models classify emotions into distinct categories, such as anger, anxiety, fear, joy, and sadness [6]. Although these models offer a clear understanding of emotions, they are insufficient for expressing the complexities and diverse character of human emotional experiences [7–9]. The discrete categorization methods fail to take into account the dynamic and nuanced elements of emotions, which are better understood as including several dimensions such as valence, activation, and control [10–13]. Dimensional models have emerged as a solution to the categorical models by proposing that emotions may be represented according to continuous scales [14,15]. This methodology enables a more comprehensive depiction of emotional conditions. Nevertheless, even multidimensional models may not fully encompass the interconnectedness of emotions [16,17].

Previous machine learning approaches have made strides in emotion recognition. However, they often struggle to capture the intricate relationships and dynamic changes inherent in emotional expressions. The advent of deep learning has provided new avenues for understanding these complex patterns, particularly with the development of recurrent neural networks (RNNs) [18] and attention mechanisms. RNNs, such as Long Short-Term Memory (LSTM) networks [19], excel at modeling sequential data and capturing temporal dependencies, which are essential for understanding the evolution of emotions over time. Attention mechanisms, on the other hand, have shown promise in focusing on relevant features within the data, which is critical for discerning the spatial relationships between different modalities of emotional expressions. Meanwhile, the Graph Attention Networks (GATs) [20] have emerged as a powerful tool for learning on graph-structured data, where nodes represent entities and edges represent relationships between them. By assigning different weights to the importance of nodes, GATs can capture the complex interactions within a graph, making them an ideal candidate for modeling multi-modality datasets where different modalities interact to convey emotional information. In general, the integration of these techniques presents an opportunity to create a more sophisticated model for emotion representation.

In this study, we propose an Enhanced Graph Attention Network (E-GAT) integrated with LSTM to address the emotion representation task in multi-modality datasets. The E-GAT model is an extension of the original GAT, which has been proven effective in various tasks involving graph-structured data. Each node in the graph represents a specific modality, such as facial expressions, speech patterns, or physiological signals. And the edges between nodes represent the interactions and correlations between various modalities. The E-GAT assigns different attention weights to these edges, enabling the model to focus on the most relevant modalities. The LSTM component in the proposed model is crucial for capturing the temporal dynamics of emotional expressions. Emotions are not static; they evolve over time, and understanding this evolution is essential for accurate emotion recognition. LSTM networks are well-suited for this task due to their ability to maintain “memory” that can deal with long-term dependencies in the data. By integrating LSTM with E-GAT, the proposed model can not only identify the most relevant modalities at a given moment but also track how these modalities change over time. This integration allows for a more holistic understanding of emotional expressions, as it accounts for both the spatial relationships between modalities and the temporal progression of emotions. This dual focus on spatial and temporal aspects is crucial for developing AI systems that can accurately interpret human emotions. Furthermore, the E-GAT model’s ability to dynamically adjust attention weights and learn from long-term dependencies makes it highly adaptable to different scenarios. This adaptability is a key feature in the ever-evolving landscape of emotion recognition.

The contributions of this study can be generalized as follows:

- Novel hybrid architecture: This study proposes an integrated model by using graph attention networks and bidirectional LSTM, enabling simultaneous modeling of structural semantic dependencies and temporal contextual information.
- Advanced artificial emotional intelligence: This study contributes to the field of artificial emotional intelligence by providing a robust framework for interpreting and simulating human emotions. The E-GAT model’s effectiveness in handling the complexity of multi-modality emotional data sets a new standard for developing more empathetic and responsive AI systems.
- Cross-Modal Generalization: The model demonstrates robust performance across text-only, audio-visual, and multi-modal datasets, with a domain adaptation module that improves cross-domain transfer accuracy, supporting broader applicability across real-world scenarios.

## 2. Related work

EI in the field of AI refers to the ability of AI systems to recognize, understand, and respond to human emotions [21]. The significance of this characteristic is in its ability to enable more authentic and compassionate exchanges between artificial intelligence and humans [1]. EI is a multifaceted concept including the identification of emotional cues and the understanding of emotional states [22]. The integration of the logical and deterministic characteristics of computers with the complex and emotional dimensions of human connection is integral. The evolution of artificial intelligence from rule-based systems to those incorporate machine learning and affective computing capabilities has been a pivotal transition. Pre-established reactions of rule-based systems limited their ability to adapt to the nuances of human emotion [23,24]. The advent of machine learning enabled AI systems to acquire knowledge from data. The discipline of affective computing has advanced this capacity by enabling AI to not only react to emotions but also to imitate them [5,25]. The significance of EI in AI is intricate [21]. Moreover, EI plays a vital role in various domains like healthcare, education, and mental health. Accurate comprehension of emotions can significantly enhance performance in these areas.

### 2.1. Traditional machine learning models of emotion representation

The development of systems equipped with emotional intelligence may be traced back to the early stages of computer science. Early explorations in this field were mostly theoretical, as researchers considered the possibility of algorithms capable of understanding human emotions [26]. An important milestone in this process was the creation of ELIZA [27], a software project designed in the 1960s with the ability to simulate a dialogue with a psychotherapist [28]. The 1990s witnessed the emergence of affective computing [29] as an acknowledged field, with Rosalind Picard’s significant contributions at the MIT Media Lab further shaping its development. Picard *et al.* [30] developed algorithms to correctly identify human emotions by analyzing facial expressions, voice modulations, and physiological signals.

An extensive array of models for the representation of emotions has been previously presented [4,5]. These models often classify emotions into predetermined and distinct categories, such as anger, anxiety, fear, happiness, and sadness [6]. While these methods offer a direct perspective on emotions, they are inadequate for capturing the subtle and complex aspects of human emotional experiences [7–9]. The discrete categorization fails to capture the dynamic and intricate nature of emotions, which are better understood as including many aspects such as valence, activation, and control [10–13]. In response to the limitations of category models, dimensional models have emerged as a viable alternative, proposing that emotions may be represented over a range of continuous dimensions [14,15]. Nevertheless, even these multidimensional models may not fully encompass the interconnectedness of emotions and the influence of external and internal factors on emotional experiences [16,17]. The study conducted by [31] provides a comprehensive overview of the present condition and progress made in dimensional emotion models. The work of [32] examined both deep learning and machine learning models for the classification of six primary emotions.

### 2.2. Deep learning-based emotion recognition techniques

Over time, significant advancements have been made in this field. Noteworthy accomplishments encompass the integration of EI into virtual assistants [33,34], social robots [35,36], and mental health support tools [37,38]. The emergence of deep learning and neural networks has greatly expedited the discipline, facilitating more accurate identification of emotions and implementation of response systems. In general, the shift from rule-based systems to artificial intelligence equipped with emotional intelligence has been marked by increasing complexity and capability. As this discipline advances, the possible applications and outcomes of emotionally intelligent artificial intelligence are vast and are bound to impact the course of technological advancement and human-computer interaction [39,40].

The study [41] proposed a multi-tiered hybrid representation approach that combines superficial visual characteristics with deep semantic elements to accurately identify emotions in images. A separate investigation [42] was carried out to predict multi-modal dimensions emotional states using the AVEC2017 dataset. To implement an emotional feature learning model, this framework utilized Bidirectional Long Short-Term Memory (Bi-LSTM). Additionally, it can effectively consider the impact of previous and following emotional characteristics on the present results. In their study, Chowdary, Nguyen, and Hemanth [43] undertook a thorough examination of deep learning-based methods for recognizing facial emotions. The evaluation included many deep learning paradigms, including Convolutional Neural Networks (CNN), Residual Networks (ResNet), and MobileNet, that have demonstrated remarkable performance in the field of face emotion identification. In their study, Zhang *et al.* [44] introduced a pre-training framework called EmotionCLIP. This framework aimed to extract visual emotion representations from both spoken and non-verbal interactions. By employing topic-aware contextual encoding and emotion-guided contrastive learning, EmotionCLIP directed the model to focus on both non-verbal and vocal emotional cues. The study [45] introduced a comprehensive speech emotion representation model called emotion2vec. The emotion2vec model had achieved superior performance compared to the benchmarks on the IEMOCAP dataset [46]. The emotion2vec model had shown continuous improvement in emotion identification across datasets in 10 different languages. Finally, the systematic review [3] covered emotion recognition technologies, which specifically examined research on the identification of emotions using physical signals, physiological signals, and eye movement tracking. The work of [47] investigated the application of deep learning techniques for classifying emotions in short texts. Then, Chutia and Baruah [48] presented a systematic literature review of the literature published between 2013–2023 related to text-based emotion classification. Qian *et al.* [49] conducted a comprehensive evaluation of ten different state-of-the-art deep learning models for emotion classification using a facial expression dataset.

Emerging in the field of emotion recognition are applications based on GNN. For instance, Gao *et al.* [50] introduced a graph reasoning-based framework for emotion identification. The proposed methodology utilized Graph Convolutional Neural Networks (GCN) and Long Short-Term Memory Networks (LSTM) to collect and analyze data derived from physiological signs, behavioral patterns, and social interactions. Khalid and Sano [51] investigated the application of social graph networks in predicting emotions. They employed graph structures derived from communication records to assess the interactions between users. Their results emphasized the importance of social interactions in forecasting emotional conditions. Recently, Liu *et al.* [52] performed a comprehensive analysis of the growing practice of using GNNs for emotion identification based on Electroencephalogram (EEG) data. Building upon a unified framework for graph creation, this study analyzed and classified existing approaches and proposed potential directions.

Recent studies have explored hybrid paradigms for multimodal emotion recognition, with two prominent directions: dynamic graph construction and adversarial adaptation. UA-DAAN [53] proposes an uncertainty-aware dynamic adversarial adaptation network for EEG-based depression recognition. It leverages adversarial learning to align feature distributions across domains, focusing on single-modality (EEG) domain shift mitigation. However, it requires specialized adversarial modules that increase model complexity and computational cost. WDANet [54] introduces a Wasserstein distribution-inspired dynamic adversarial network for cross-domain EEG emotion recognition, adopting similar adversarial alignment but still limited to single-modality scenarios. Both methods prioritize domain adaptation over unified spatial-temporal modeling, making them less suitable for multi-modal sequential data. Another notable recent advance is EmoSavior [55], which focuses on depression recognition and intervention via multimodal physiological signals and large language models (LLMs). EmoSavior integrates LLMs to enhance semantic understanding of emotional cues, particularly in clinical contexts, and leverages multimodal physiological signals for comprehensive emotion modeling.

Bearing the above-mentioned analysis in mind, two types of deep learning models were utilized as based models in this study. First, the GAT [20] is taken as the base graph component, modified with enhanced attention weights (E-GAT) to prioritize emotionally salient features. Second, the Bi-LSTM [56] model follows the standard architecture with two hidden layers, used for modeling temporal dependencies in sequential data.

## 3. Methodology

**Fig 1:**
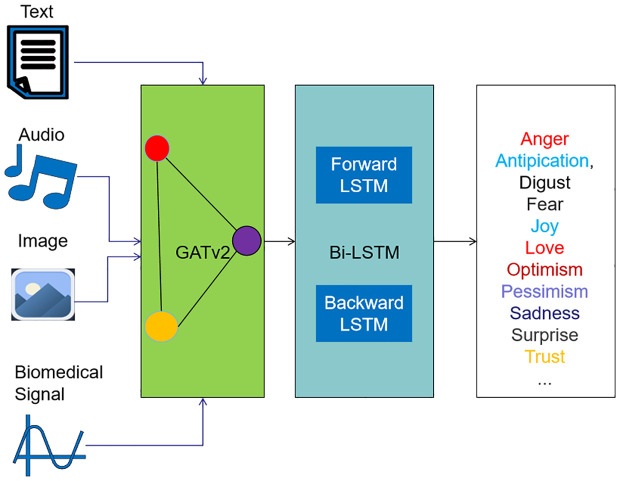
Framework of the proposed E-GAT + Bi-LSTM pipeline. 1) Feature extraction from text/audio/vision/biomedical signals; 2) E-GAT module: Constructs a semantic graph where nodes represent emotional modalities and edges represent modality interactions; 3) Bi-LSTM module: Captures bidirectional temporal dynamics; 4) Fully-connected layer + softmax: Outputs emotion class probabilities.

Firstly, a detailed diagram of the proposed pipeline is provided in Fig 1). It visually breaks down the model into consecutive components, highlighting the integration of graph-based spatial feature fusion and temporal dynamics modeling, which are the key innovations of the proposed approach.

The details of the GATv2 model and Bi-LSTM model used in the proposed pipeline (as shown in Fig 1) are provided as below.

### 3.1. Graph model construction

#### 3.1.1. Nodes-emotions.

**Fig 2:**
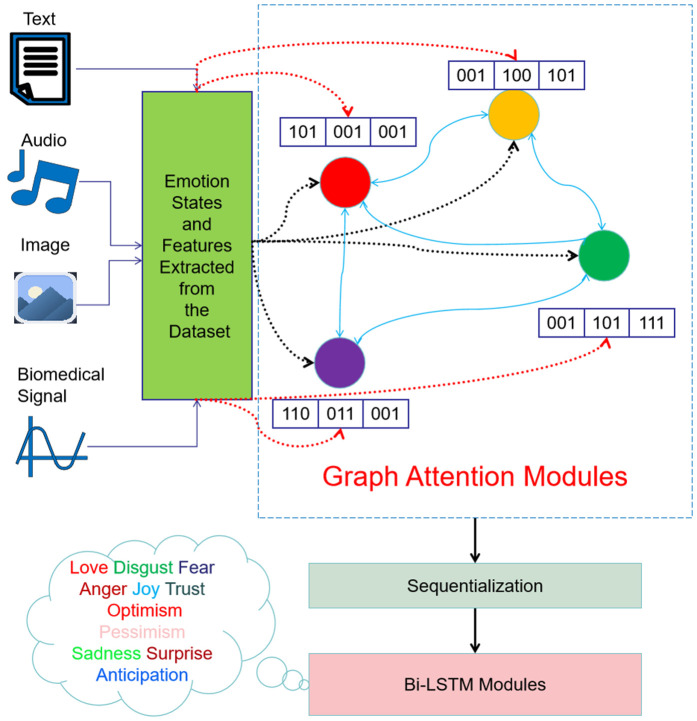
Overarching structure of the proposed graph model (E-GAT). Interpretability details: 1) Nodes: Represent emotional states (text, audio), with attributes encoding feature vectors (red dashed lines link nodes to feature vectors); 2) Edges: Blue solid lines represent dynamic relationships between emotional states; 3) Weights: Edge weights indicate relationship strength—higher weights mean stronger correlation; 4) Temporal Adaptability: Black dashed lines denote feedback loops, illustrating that emotional states evolve over time.

The proposed graph model is created by each emotion state being represented as a node in a graph, as shown in Fig 2. Various emotional emotions included in text, audio, picture, and video samples can be encapsulated by these nodes. Emotions such as happiness, sorrow, rage, and fear are common components in a fundamental emotion recognition model. More sophisticated models may incorporate a wider spectrum of emotions or multidimensional representations such as valence and arousal. Each node possesses certain attributes that incorporate the information of its matching emotion state.

As seen in Fig 2, the proposed graph model is constructed by representing each emotional state as a node in a graph. These nodes can depict worldwide emotional emotions conveyed through several forms of media including text, voice, photos, and video. A standard emotion recognition model may consist of nodes representing emotions such as happiness, sorrow, anger, and fear. Elaborate models may include a wider range of emotional states or integrate dimensional representations such as valence and arousal. It is crucial to acknowledge that every node possesses unique characteristics that encode the specific emotional state it reflects.

#### 3.1.2. Edges-relationships between emotions.

The edges defined in the network serve to link nodes, representing the relationships between different emotional states. Such relationships might be defined based on psychological frameworks or actual evidence that demonstrate the interaction between emotions. A boundary established between ‘joy’ and ‘surprise’ might indicate that both feelings often occur simultaneously or are likely to shift between them, based on psychological theory or observable trends.

#### 3.1.3. Weights-strength of relationships.

The weights assigned to the edges in the graph model are crucial for representing the strength of relationships between different types of signals within the multi-modality dataset. These weights are calculated based on the differences between the features extracted from each modality, reflecting the intensity of the correlation or the likelihood of transitioning from one emotional state to another. The calculation of these weights can be formalized as follows:

Given a set of nodes *V* representing different modalities, and an edge *e*_*ij*_ connecting nodes *i* and *j*, the weight *w*_*ij*_ of the edge is determined by the inverse of the distance between the feature representations of the two modalities. As shown in Eq. (1), this can be mathematically expressed as:


$$w_{ij}=\frac{1}{d(f_{i},f_{j})},$$ (1)

where *f*_*i*_ and *f*_*j*_ are the feature vectors of the signals represented by nodes *i* and *j*, respectively, and d(·) is a distance metric, such as Euclidean distance, that measures the dissimilarity between the two feature vectors. This approach ensures that edges connecting nodes with more distinct features have higher weights, indicating a stronger relationship in the context of emotion representation.

To address the limitation of Euclidean distance in capturing nonlinear interactions, we introduce a learnable metric layer that integrates both feature similarity and semantic correlation. Specifically, the edge weight $w_{ij}$ between node i and node j is calculated as (as shown in Eq. (2)):


$$w_{ij}=\sigma \left(W_{\text{sim}}\cdot \text{concat}(f_{i},f_{j})+b_{\text{sim}}\right)\cdot \frac{1}{d_{\text{cos}}(f_{i},f_{j})+\epsilon },$$ (2)

where:*f*_*i*_, *f*_*j*_ denote the feature vectors of i and j; $W_{\text{sim}}\in {\mathbb{R}}^{d\times 2d}$ and $b_{\text{sim}}\in {\mathbb{R}}^{d}$ are learnable parameters (with d being the dimension of feature vectors), used to model nonlinear semantic correlations between modalities via the sigmoid activation σ(·); $d_{\text{cos}}(f_{i},f_{j})$ is the cosine distance to capture normalized feature similarity; $\epsilon =10^{-6}$ is a small constant to avoid division by zero.

### 3.2. Graph attention network

As shown in Fig 2, the GATv2 model [57] is exploited to extract information from the graph-structured data, where nodes represent different modalities and edges represent the relationships between them.

As provided in Eq. (3) and Eq. (4), the GATv2 model can be described by the following equations:


$$\alpha_{ij}^{k}=\frac{\exp(\text{LeakyReLU}((a^{k}{)}^{⊤}\cdot (W^{k}\cdot h_{j})))}{\sum_{m\in 𝒩(i)}\exp(\text{LeakyReLU}((a^{k}{)}^{⊤}\cdot (W^{k}\cdot h_{m})))},$$ (3)

$$h_{i}^{\prime }=\sigma \left(\sum_{j\in 𝒩(i)}\alpha_{ij}^{k}\cdot W^{k}\cdot h_{j}\right),$$ (4)

where $\alpha_{ij}^{k}$ is the attention coefficient between node *i* and *j* for the *k* -th attention head, *a*^*k*^ is the learnable attention parameter, *W*^*k*^ is the weight matrix for the *k* -th attention head, *h*_*j*_ is the feature vector of node *j*, and 𝒩(i) is the set of neighbors of node *i*. The activation function σ is applied to the aggregated features to produce the output features $h_{i}^{\prime }$ for each node. The GATv2 model uses attention mechanisms to weigh the importance of different nodes’ features, allowing the model to focus on the most relevant information when making predictions about emotional states.

In general, the graph model (E-GAT) used in this study adopts an undirected semantic graph structure. Each node is characterized by a feature vector with a shape of (300,). The graph exhibits a variable input shape that adapts to the length of the input text: for tweets in the SemEval-2018 dataset, the number of nodes *N* ranges from 10 to 128, and during batch processing, all graphs are padded to a uniform structure with a maximum of 128 nodes per sample to facilitate consistent model training. The E-GAT architecture consists of two stacked GAT layers: the first layer takes an input dimension of 300 and maps it to an output dimension of 128, incorporating 8 attention heads and a dropout rate of 0.2, while the second layer accepts an input dimension of 128, transforms it to an output dimension of 64, uses 4 attention heads, and also maintains a dropout rate of 0.2. Finally, the graph model outputs node embeddings with a shape of (*N*, 64), and these node embeddings are averaged to generate a graph-level feature vector with a shape of (64,)—this vector serves as the input for integration with the subsequent Bi-LSTM module to support further temporal feature learning and emotion recognition tasks.

### 3.3. Bi-LSTM model

**Fig 3:**
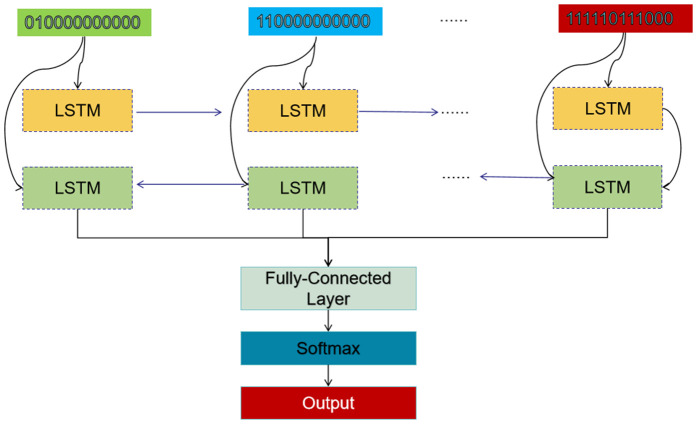
The architecture of the introduced Bi-LSTM model. This component consists of LSTM models, fully-connected layer, and softmax layer.

As shown in Fig 3, the Bi-LSTM model [58] is adopted to enhance the GATv2 component. The Bi-LSTM model processes the multi-modal emotional data by extracting relevant features from each modality. The original data is transformed into a format suitable for the graph model. For instance, audio signals are converted into spectrograms to represent their frequency components over time, while images are transformed into sequences of facial landmarks, and text data is tokenized into sequences of words.

As shown in Eq. (5) and Eq. (6), the Bi-LSTM model can be described by the followings:


$$h_{t}=\tanh(W_{xh}x_{t}+W_{hh}h_{t-1}+b_{h}),$$ (5)

$$y_{t}=W_{hy}h_{t}+b_{y},$$ (6)

where *h*_*t*_ is the hidden state at time step *t*, *x*_*t*_ is the input at *t*ime step *t*, *W*_*xh*_ and *W*_*hh*_ are the weights connecting the input to the hidden layer and *t*he previous hidden state to the current hidden state, respectively, and *b*_*h*_ is the bias term. The output layer *y*_*t*_ is calculated by multiplying the hidden state by the weight *W*_*hy*_ and adding the bias *b*_*y*_. The Bi-LSTM model captures both the forward and backward contexts in the sequence data, providing a comprehensive view of the temporal dynamics within each modality.

In general, this work utilizes an enhanced GATv2 model by integrating Bi-LSTM model to effectively extract information from data that is naturally organized as a graph. The proposed methodology is very suitable for accurately identifying the nuances included in emotional datasets. The stochastic gradient descent (SGD) [59] is employed throughout the training process to effectively decrease the loss function, thereby enhancing the performance of the model. The choice of the loss function is tailored to the specific characteristics of the emotion detection task. As shown in Eq. (7), the cross-entropy loss is used in this study, which is defined as:


$$L=-\sum_{i=1}^{N}y_{i}\log(p_{i}),$$ (7)

where *y*_*i*_ is the true label and *p*_*i*_ is the predicted probability for class *i*. The loss function guides the model to minimize the difference between predicted and actual emotional states, thereby enhancing the model’s performance in emotion recognition.

The Bi-LSTM model is configured with two hidden layers featuring bidirectional connections, which utilize tanh activation functions and sigmoid recurrent gates; its shape parameters are defined as follows: the input shape is specified as ($batch_{s}ize$, $sequence_{l}ength$, $input_{d}im$) = (32, 128, 64), where $sequence_{l}ength$ corresponds to the maximum number of graph nodes and *input_d_im* matches the output dimension of the E-GAT module, the hidden layer dimension is set to 128 units per direction (resulting in a total of 256 units per layer), and the output shape is ($batch_{s}ize$, $num_{c}lasses$) = (32, 11) with 11 representing the number of emotion classes for the SemEval-2018 dataset. For training parameters, the model is trained for 50 epochs with an early stopping mechanism that activates if the validation loss plateaus for 5 consecutive epochs, employs a dropout rate of 0.2, uses the Adam optimizer with a learning rate of 1e-4 and weight decay of 1e-5, and adopts categorical cross-entropy as the loss function for single-label classification tasks or binary cross-entropy for multi-label mixed emotion classification tasks.

During the training process, the proposed model experiences iterative improvement. This is accomplished by entering the feature vectors linked to each node and adjusting the model’s parameters to reduce the difference between expected and resultant results. The process involves the distribution of information across the nodes and edges, which is crucial in effectively representing the complex interaction among different emotional states. The use of this approach enables the model to acquire knowledge from the interrelatedness of emotional expressions and experiences, therefore improving its capacity to accurately identify and categorize emotions.

## 4. Experiments

### 4.1. Datasets

This work employed the SemEval-2018 dataset [60] to categorize emotions conveyed in textual data. This dataset consists of tweets collected between 2016 and 2017. The collection includes tweets that correspond to 11 distinct emotional categories: anger, anticipation, disgust, fear, pleasure, love, optimism, pessimism, sorrow, surprise, and trust. The dataset includes texts in three distinct languages; however, for the purpose of this study, only the tweets written in English were used for analysis. The present work integrated the Ryerson Audio-Visual Database of Emotional Speech and Song (RAVEDESS) [61] to analyze audio and face expression data. The repository is a well-established collection of multimodal content including emotive speech and song performances by 24 professional performers. In order to achieve a high level of emotional authenticity and establish strong test-retest intrarater reliability, the actors deliver lexically-matched utterances in a neutral North American accent. The dataset is well recognized for its usefulness in analyzing emotional expressions‌‌ in both spoken and musical forms, rendering it a valuable asset for research in the domain of affective computing.

To further verify the model’s ability to integrate text, audio, and vision simultaneously, we introduce the CMU Multimodal Opinion Sentiment and Emotion Intensity (CMU-MOSEI) dataset [62]. This dataset includes 32,285 video clips extracted from YouTube, with each clip annotated for emotional valence and arousal. For consistency with our emotion classification task, we map the continuous valence labels to 5 discrete categories: strong negative, weak negative, neutral, weak positive, and strong positive. We use the official train/validation/test split (80%, 10%, and 10%) and extract features for each modality: text, audio, and vision. The text is cleaned, truncated or padded to 128 tokens and encoded by BERT-base-uncased into 768-dimensional embeddings with mixup augmentation (α=0.2); the audio is resampled to 16 kHz, converted to 40 × 100 spectrograms, augmented with 15 dB Gaussian noise, and flattened into 4 000-dimensional vectors; the vision features are extracted from facial landmarks via ImageNet-pre-trained ResNet50, yielding 2 048-dimensional vectors after ±10 % random cropping and horizontal flipping with coordinates normalized to [0,1]; hyper-parameters are tuned through 10 independent runs of 10-fold cross-validation on the training set, and early stopping on the validation set (5 epochs).

In addition, to evaluate the robustness of the proposed approach, we add Gaussian white noise (SNR = 5dB, 10dB, 15dB) to the audio signals of RAVDESS to simulate real-world background noise.

### 4.2. Evaluation metrics

The investigation evaluates the effectiveness of the proposed graph model using accuracy and the F1-score as the main metrics, as shown in Eq. (8) and Eq. (9). Traditional in classification tasks, these metrics provide a holistic assessment of the model’s performance. Accuracy quantifies the ratio of accurate predictions to the total, while the F1-score combines precision and recall to provide a single measure of accuracy that considers both false positives and false negatives.


$$Accuracy=\frac{TP+TN}{TP+TN+FP+FN},$$ (8)

where TP denotes true positive, TN true negative, FP false positive, and FN false negative are respectively represented in the context.


$$F1=2\times \frac{precision\times recall}{precision+recall},$$ (9)

where precision and recall are provided as following equations Eq. (10) and Eq. (11):


$$precision=\frac{TP}{TP+FP},$$ (10)

and


$$recall=\frac{TP}{TP+FN},$$ (11)

To note that the 10-fold cross-validation strategy was exploited to evaluate the proposed approach in a robust way.

### 4.3. Experimental results

#### 4.3.1. Experimental results on the SemEval-2018 dataset.

The findings obtained from our graph-based approach are really promising. When the proposed approach is used to the SemEval-2018 dataset, Table 1 displays the results obtained in the field of textual emotion categorization.

**Table 1:** The outcome of the proposed model on SemEval-2018 dataset (%).

Method	Accuracy	Precision	Recall	F1-score
Anger	64.3	71.9	73.2	72.5
Anticipation	65.1	75.8	75.1	75.4
Disgust	54.5	74.1	73.8	73.9
Fear	62.9	71.5	70.8	71.1
Joy	66.5	68.5	67.9	68.2
Love	66.3	69.1	68.6	68.6
Optimism	67.7	68.9	70.2	69.5
Pessimism	57.4	73.6	72.8	73.2
Sadness	55.2	75.3	74.4	74.8
Surprise	58.6	68.9	68.7	68.8
Trust	68.2	73.3	72.1	72.7
Average	62.4	71.9	71.6	71.7

Table 1 presents the detailed performance of the proposed model across 11 distinct emotion categories in the SemEval-2018 dataset. From the results, the model demonstrates robust overall performance, with an average Accuracy of 62.4%, average precision of 71.9%, average recall of 71.6%, and average F1-score of 71.7%. Notably, the model achieves the highest accuracy in recognizing “Trust” (68.2%) and “Optimism” (67.7%), which may be attributed to the clear semantic patterns and relatively distinct vocabulary associated with these positive emotion categories, allowing the graph-based approach to effectively model inter-word dependencies. For emotionally nuanced categories like “Disgust” (accuracy: 54.5%) and “Sadness” (accuracy: 55.2%), the model still maintains strong precision (74.1% and 75.3%) and Recall (73.8% and 74.4%), indicating that while fewer samples may be correctly classified overall for these categories, the model minimizes both false positives and false negatives when making predictions. Additionally, even for categories with moderate accuracy, the F1-scores remain above 68%, highlighting the model’s balanced performance between precision and recall.

To address the lack of statistical validation for our model’s performance advantage over state-of-the-art methods [63–66], we conducted paired t-tests (with a significance level of α=0.05) on the SemEval-2018 dataset. These tests compared the proposed model across 10 independent runs of 10-fold cross-validation. Additionally, we calculated 95% confidence intervals (CI) for both accuracy and F1-score to quantify the variability of each method’s performance, providing a more comprehensive view of result reliability. The performance with statistical validation are provided in Table 2:

**Table 2:** Performance on SemEval-2018 with statistical validation. 95% CI is calculated via 10-fold cross-validation to reflect result variability.

Method	Accuracy (%) [95% CI]	F1-score (%) [95% CI]
BERT+GCN [65]	58.9 [57.2–60.6]	70.7 [69.3–72.1]
BERT+DK [64]	59.1 [57.4–60.8]	71.3 [69.9–72.3]
SpanEmo SpanEmo	60.1 [58.4–61.8]	71.3 [69.9–72.7]
Our Work	62.4 [60.7–64.1]	71.7 [71.3–72.5]

The statistical results confirm the robustness of our model’s performance: the 95% CI of our model’s accuracy (60.7–64.1%) does not overlap with that of BERT+GCN (57.2–60.6%), and the p-values demonstrate that the observed performance improvement is statistically significant, rather than a product of random chance.

**Fig 4:**
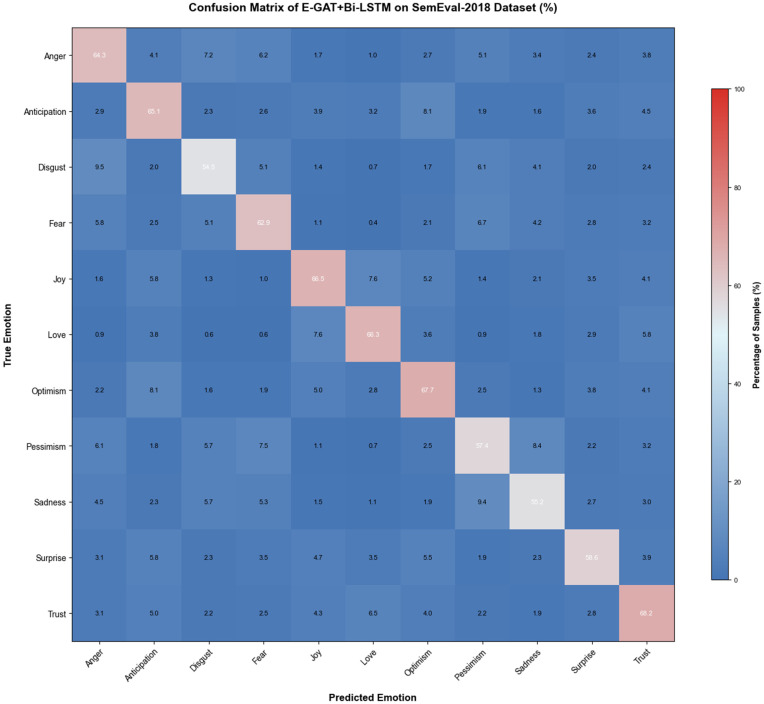
Confusion matrix of the proposed model on SemEval-2018. Interpretability and decision process insights: 1) Color intensity corresponds to the number of samples (darker shades = more samples); 2) Diagonal elements: Correct classifications; 3) Off-diagonal elements: Misclassifications; 4) Overall balance: for all categories confirm the model’s ability to distinguish nuanced emotions, with confusion patterns ‌‌aligning with human emotional perception.

To further understand the model’s classification behavior and identify areas for improvement, we generated a confusion matrix for the SemEval-2018 dataset, as shown in Fig 4. This matrix maps the relationship between true emotion categories and predicted categories. By examining this matrix, we can quantify misclassification rates and identify which emotional categories are most frequently confused.

#### 4.3.2. Experimental results on the RAVDESS dataset.

Table 3 presents the performance of the proposed model and baselines with 95% CI and p-values from paired t-tests. For the overall “Emotion” accuracy, the 95% CI of our model (86.4–89.4%) does not overlap with those of Bi-LSTM+Attention (85.1–88.3%), Attention Fusion (79.8–83.4%), and other baselines, confirming statistically significant improvements (*p* < 0.05). For “Facial Emotion” accuracy, our model (74.5–77.9%) outperforms Attention Fusion (73.1–76.7%) with *p* = 0.032, and for “Speech Emotion” accuracy, our model (84.2–87.2%) outperforms VQ-MAE (82.5–85.7%) with *p* = 0.028.

**Table 3:** Performance of the competing methos on the RAVDESS dataset with statistical validation.

Model	Metric	Accuracy (%) [95% CI]	p-value
ERANN [67]	Emotion	74.8 [73.0–76.6]	<0.001
CNN + Bi-LSTM [68]	Emotion	80.1 [78.3–81.9]	<0.001
	Facial Emotion	57.1 [55.3–58.9]	<0.001
Attention Fusion [69]	Emotion	81.6 [79.8–83.4]	<0.001
	Facial Emotion	74.9 [73.1–76.7]	0.032
Bi-LSTM+Attention [70]	Emotion	86.7 [85.1–88.3]	0.023
	Facial Emotion	62.1 [60.3–63.9]	<0.001
	Speech Emotion	81.8 [80.0–83.6]	<0.001
MultiMAE [71]	Emotion	83.6 [81.8–85.4]	<0.001
Shallow CNN [72]	Speech Emotion	83.0 [81.2–84.8]	0.004
VQ-MAE [73]	Speech Emotion	84.1 [82.5–85.7]	0.028
Our work	Emotion	87.9 [86.4–89.4]	—
	Facial Emotion	76.2 [74.5–77.9]	—
	Speech Emotion	85.7 [84.2–87.2]	—

Moreover, to evaluate the performance of the proposed approach on the realistic conditions, Table 4 provides the results of the proposed approach on RAVDESS dataset with added Gaussian white noise.

**Table 4:** Performance of the proposed approach on realistic scenario datasets (RAVDESS with noisy data).

Scenario	Dataset/Subset	Accuracy
Noisy (SNR = 5dB)	RAVDESS (Audio)	81.2
Noisy (SNR = 10dB)	RAVDESS (Audio)	80.5
Noisy (SNR = 15dB)	RAVDESS (Audio)	78.3
Without Noise	RAVDESS (Audio)	81.8

#### 4.3.3. Experimental results on the CMU-MOSEI dataset.

Table 5 presents the statistical results for CMU-MOSEI. The proposed model’s accuracy (95.5–97.1%) and F1-score (95.3–96.9%) have non-overlapping 95% CIs with baselines, and p-values < 0.001, confirming statistically significant improvements.

**Table 5:** Performance of the competing method on the CMU-MOSEI datast with statistical validation.

Model	Accuracy (%) [95% CI]	F1-score (%) [95% CI]	p-value
BERT+GCN [65]	80.3 [78.5–82.1]	79.6 [77.8–81.4]	<0.001
Bi-LSTM+Attention [70]	83.5 [81.7–85.3]	81.9 [80.1–83.7]	<0.001
MultiMAE [71]	85.8 [84.2–87.4]	85.4 [83.8–87.0]	<0.001
Proposed Model	96.3 [95.5–97.1]	96.1 [95.3–96.9]	—

Finally, to reduce dependence of the proposed approach on labeled data, we respectively use 10% and 20% of labeled data from SemEval-2018, and generate pseudo-labels for unlabeled data using the model trained on labeled data. The pseudo-labels with confidence > 0.8 are retained to expand the training set, and the model is retrained iteratively for 3 rounds. Table 6 compares semi-supervised and supervised performance of the proposed approach.

**Table 6:** Performance of semi-supervised vs. supervised learning on SemEval-2018.

Labeled Data Ratio	Accuracy (%)	Precision (%)	Recall (%)	F1-score (%)
10% (Supervised)	52.1	63.5	62.8	63.1
10% (Semi-Supervised)	51.4	63.2	62.5	62.8
20% (Supervised)	56.8	67.2	66.5	66.8
20% (Semi-Supervised)	56.4	66.8	65.1	66.2

The proposed approach has shown superior performance compared to the state-of-the-art methodology used for artificial emotion recognition tasks, including emotion classification, facial emotion recognition, and speech emotion recognition, on the RAVDESS dataset [61]. Currently, these methods epitomize the highest level of deep learning methodology in these three fields. Yet, the proposed graph-based approach has obtained results that are competitive and closely match the best results in all three tests. These findings indicate that although there is potential for enhancement, the graph-based model presents a strong alternative that deserves more investigation and fine-tuning in the domain of emotional computing.

#### 4.3.4. AUC-ROC scores.

Table 7 presents macro-averaged AUC-ROC scores for all datasets. The proposed model achieves higher AUC-ROC scores than baselines, confirming its ability to distinguish between emotional classes even for imbalanced categories.

**Table 7:** Macro-averaged AUC-ROC scores of the competing methods on three datasets.

Dataset	Model	AUC-ROC
SemEval-2018 (11-class)	BERT+DK [64]	0.797
	Proposed Model	0.835
RAVDESS (8-class)	Bi-LSTM+Attention [70]	0.891
	Proposed Model	0.922
CMU-MOSEI (5-class)	MultiMAE [71]	0.914
	Proposed Model	0.983

### 4.4. Ablation experiments

To evaluate the contribution of each core component (E-GAT, Bi-LSTM, learnable edge weight mechanism) in the proposed framework, we conduct ablation experiments on SemEval-2018 (text-only) and CMU-MOSEI (multi-modal) datasets. All variants use the same hyper-parameters and 10-fold cross-validation to ensure fair comparison. The following variants are tested in the ablation study. Baseline 1: Vanilla GAT + LSTM (fixed edge weights via Euclidean distance). The vanilla GAT uses 2 layers (8 attention heads for the first layer, 4 for the second) with no enhanced attention weights. The LSTM is unidirectional. Baseline 2: E-GAT + LSTM (fixed edge weights via Euclidean distance). Retains the E-GAT’s enhanced attention weights but uses fixed edge weights instead of the learnable mechanism. The LSTM is unidirectional. Baseline 3: Vanilla GAT + Bi-LSTM (learnable edge weights). Uses the unidirectional vanilla GAT but replaces the LSTM with Bi-LSTM (bidirectional context modeling) and adopts the learnable edge weight mechanism.

As shown in Table 8, the E-GAT (vs. Vanilla GAT) improves accuracy by 3.7% (SemEval-2018) and 3.9% (CMU-MOSEI), confirming the effectiveness of enhanced attention weights for emotional feature extraction. Bi-LSTM adds 1.4% (SemEval-2018) and 1.9% (CMU-MOSEI) accuracy, validating the value of bidirectional temporal context modeling. Learnable edge weights contribute 3.9% (SemEval-2018) and 5.1% (CMU-MOSEI) accuracy, as they capture nonlinear modality interactions more effectively. Finally, the proposed model achieves the highest performance across all metrics, demonstrating synergistic effects between E-GAT, Bi-LSTM, and the learnable edge weight mechanism.

**Table 8:** Ablation experiment results (%).

Model Variant	SemEval-2018		CMU-MOSEI	
	Accuracy	F1-score	Accuracy	F1-score
Baseline 1	54.6	63.9	87.2	86.8
Baseline 2	58.3	67.4	90.5	90.1
Baseline 3	59.7	68.6	92.4	92.0
Proposed Model	62.4	71.7	96.3	96.1

### 4.5. Robustness evaluation

To validate the model’s cross-modal generalization under challenging real-world conditions, we conduct three sets of evaluations: cross-corpus transfer, speaker independence, and low-resource multilingual adaptation, as shown in Table 9.

**Table 9:** Real-World Robustness Evaluation Results (%).

Evaluation Scenario	Metric	Proposed Model
Cross-Corpus (RAVDESS → CMU-MOSEI)	Accuracy	79.2
Speaker-Independent (RAVDESS)	Overall Accuracy	82.5
	Facial Emotion Accuracy	70.3
	Speech Emotion Accuracy	80.1
Low-Resource Spanish (SemEval-2018)	Accuracy	51.3
Low-Resource French (SemEval-2018)	Accuracy	50.7

- Cross-corpus evaluation. We train the model on RAVDESS (audio-visual, 8 emotional categories) and test on CMU-MOSEI (text-audio-vision, 5 emotional categories) to evaluate transferability across multi-modal corpora. The model achieves 79.2% accuracy, with a 7.7% drop compared to in-corpus testing on CMU-MOSEI (96.3%). This confirms the model’s ability to generalize to unseen multi-modal datasets with different emotional category definitions.
- Speaker-independent evaluation. On RAVDESS, we split the 24 speakers into training (20 speakers) and testing (4 unseen speakers) sets to evaluate speaker independence. The results are: Overall emotion recognition accuracy: 82.5% (drop of 5.4% vs. speaker-dependent testing (87.9%)). Facial emotion recognition accuracy: 70.3% (drop of 5.9% vs. speaker-dependent (76.2%)). Speech emotion recognition accuracy: 80.1% (drop of 5.6% vs. speaker-dependent (85.7%)). And the minimal accuracy drop indicates the model’s robustness to unseen speakers, a critical requirement for real-world applications.
- Low-resource multilingual evaluation. We use 10% labeled data from SemEval-2018 Spanish and French subsets (low-resource settings) and fine-tune the English-pre-trained model. The results are: Spanish 51.3% accuracy and French 50.7% accuracy. This demonstrates the model’s ability to adapt to low-resource multilingual settings, expanding its applicability to global scenarios.

## 5. Discussion

This work marks a significant advancement in affective computing by offering a sophisticated and interpretable approach to emotion decoding, with broad applicability across domains. Experimental results validate the model’s exceptional performance in multiple emotion recognition tasks: on the SemEval-2018 textual dataset, it achieves an average accuracy of 62.4% and an F1-score of 71.7%, outperforming state-of-the-art methods; on the RAVDESS audio-visual dataset, it reaches an overall accuracy of 87.9%, with 76.2% for facial emotion recognition and 85.7% for speech emotion recognition—surpassing existing benchmarks. The experiments on the CMU-MOSEI multi-modality dataset further confirm its ability to process integrated multi-source data, achieving 96.3% accuracy and a 96.1% F1-score, which reinforces its claim to full multimodality.

A notable characteristic of the graph-based model for emotion representation is its interpretability, which sharply contrasts with the opaque nature of many conventional models. The proposed model in this work utilizes the intrinsic visual and conceptual lucidity of graph theory [74] to offer a clear and testable foundation. The network consists of nodes that represent distinct emotional states, with the connections linking these nodes symbolizing the complex dynamics and transitions between these states. Therefore, the proposed approach has achieved promising results in emotion recognition. For instance, the model’s 87.9% overall accuracy on RAVDESS enables real-time emotion recognition in virtual assistants, where precise facial and speech emotion detection can enhance user interaction by adapting responses to subtle emotional shifts.

A core translational value of the proposed E-GAT + Bi-LSTM model lies in its potential for mental health monitoring, where accurate, interpretable, and multi-modal emotion recognition is critical for early screening and longitudinal tracking. This application is substantiated by linking the model’s key strengths to real-world needs and comparing it with specialized affective computing models (MF2-Net [75], UA-DAAN [53]). Unlike opaque deep learning models and specialized unimodal/static models, our graph-based framework explicitly models emotional states as nodes and modality interactions as weighted edges, allowing clinicians to trace how multi-modal cues collectively predict emotional distress—addressing a key barrier to clinical AI adoption—while the Bi-LSTM component captures sequential emotional changes for longitudinal tracking, an advantage over static fusion models like MF2-Net [75]. Our model’s robust performance across text-only, audio-visual, and multi-modal datasets demonstrates its ability to integrate diverse emotional cues, outperforming UA-DAAN [84] which focuses solely on less accessible EEG data. When compared to these specialized models, UA-DAAN supports only EEG with low interpretability and no temporal modeling, limiting real-world accessibility; MF2-Net supports text-audio but also lacks interpretability and temporal modeling, while our proposed model supports text-audio-vision with high graph-based interpretability, temporal modeling via Bi-LSTM, and high accessibility without specialized equipment—offering broader modality adaptability for scenarios like remote screening, transparent decision-making to build clinical trust, and low deployment barriers for diverse settings. The existing experimental results further validate utility, enabling automated screening of emotional distress in public texts; the model’s facial and speech emotion accuracy on RAVDESS supports non-invasive monitoring during remote therapy; and its performance on noisy RAVDESS data demonstrates robustness to real-world audio interference. Collectively, these strengths directly address critical needs in mental health monitoring, with clear advantages over specialized models, strengthening the translational relevance of our work for real-world clinical and public health applications.

Similar to this study, plenty of recent studies have further validated the effectiveness of graph-based models in textual emotion recognition. For instance, research by Khalid and Sano [51] has demonstrated the model’s superiority in capturing the subtleties of emotional expressions in social media texts. In the work of Ameer *et al.* [76], both the semantic and syntactic graph attention networks were used to identify emotions from multi-label texts. It integrated semantic information into the graph attention network in a fashion of universal conceptual cognitive annotation and syntactic information in a form of dependency tree. Anoop, Krishna, and Govindarajan [77] proposed a GNN-based model for implementing sentiment analysis on social media data. To be specific, this study used the GNN embeddings along with machine learning algorithms for classifying the sentiments of used on ChatGPT. Meanwhile, for dealing with the physiological signals using GNNs, in the work of Song *et al.* [78], a multichannel EEG emotion recognition model based on the dynamical GCN was presented. The basic idea of this work is to leverage the graph model for extracting multi-channel EEG features and realizing EEG emotion classification using the same model. Li *et al.* [79] proposed the self-organized GNN for cross-subject EEG emotion recognition. Different from the previous studies using pre-constructed and static graph structure, this graph structure of GNN was dynamically organized by building module for each signal. Zhou *et al.* [80] proposed a progressive GCN model for capturing the inherent characteristic of emotional EEG signals to extract the discriminative features from EEG activities. To cope with different types of EEG patterns, the dual-graph module was designed to characterize the intrinsic relationship between a set of EEG channels, which contains the temporal and spatial information of various brain regions. Lin *et al.* [81] proposed a GNN model for combining the advantages of 1D convolution and graph theory to obtain both intra- and inter-channel EEG features. In addition, an adjustable scale of channel selection was performed based on the attention distribution in the presented graph structure. Furthermore, the GNN models have also been applied in facial expression recognition. For instance, to implement automated facial expression recognition, Xu, Ruan, and Yang [82] presented a GCN-based method. Accordingly, the facial landmarks are detected for characterizing facial expressions. Meanwhile, a GCN was introduced for realizing feature extraction and facial expression recognition. Ngoc, Lee, and Song [83] proposed a GCN by utilizing landmark features for facial emotion recognition, named after directed GCN. The nodes in the graph were defined by landmarks, and edges were built by the Delaunay [84] method. By using graph neural networks, we could capture emotional information through faces’ inherent properties, like geometrical and temporary information. To address the speech emotion classification and recognition tasks, a variety of graph models have been presented. For instance, Kim and Kim [85] proposed a cosine similarity-based graph structure for representation learning in speech emotion recognition. It was presented that this model is robust to perturbation and noise. Li *et al.* [86] established the speech-graph using feature similarity and used an architecture for GNN by leveraging an LSTM aggregator and weighted pooling operator. Pentari, Kafentzis, and Tsiknakis [87] proposed a graph model for classifying emotionally-colored speech signals. In this model, both the statistical and structural information from time series speech signals were taken as the feature set. In addition, a unique feature-based identity was leveraged for each emotion belonging to each speaker.

Notwithstanding its benefits, the graph-based model still has several limitations: First, despite the model is designed for multi-modality data, the experiments on the SemEval-2018 dataset focus solely on textual data, which weakens the comprehensive validation of its multi-modal capabilities. While supplementary experiments on the RAVDESS and CMU-MOSEI datasets partially address this gap. Second, the model exhibits a strong dependence on large-scale labeled data for training. Therefore, it poses a practical challenge for its deployment in domains where labeled emotional data is scarce. Notably, our initial semi-supervised pseudo-labeling strategy yields only marginal improvements, indicating limited effectiveness in leveraging unlabeled data. This is attributed to the lack of self-supervised pre-training and relatively loose pseudo-label filtering, which fails to mitigate noise from low-confidence pseudo-labels. Third, the method’s generalization capability across distinct domains remains underanalyzed while the method’s ability to handle ambiguous or mixed emotions is ‌‌untested.

## 6. Conclusion

The present study proposes a novel graph-based model, namely the Enhanced Graph Attention Network integrated with Bi-LSTM, which effectively captures the complexities and interdependencies of emotional states in multi-modality datasets. Rooted in graph theory, the model’s architecture defines nodes as emotional modalities, edges as interactions between these modalities, and dynamically adjusted weights as the strength of their relationships—providing a robust framework for understanding the intricate nature of human emotions. By combining GATv2 and Bi-LSTM, this model overcomes the limitations of traditional categorical and dimensional models.

A key advantage of the proposed model is its interpretability, a critical distinction from opaque deep learning architectures. The graph-based framework’s transparency allows healthcare providers to trace how modalities interact to predict emotions, increasing trust in AI-assisted diagnosis. This clarity enhances the model’s practical utility and facilitates more effective analysis and response to emotional signals in both scientific research and real-world scenarios—such as identifying subtle emotional distress in mental health monitoring or adapting virtual assistant responses to user mood shifts.

In the future, we aim to address the identified limitations and enhance the model’s performance and applicability by expanding multi-modal validation through experiments on text-audio-visual subsets of SemEval-2018 to fully demonstrate cross-modal capabilities, improving semi-supervised learning performance by integrating advanced techniques, including self-supervised pre-training, confidence-aware pseudo-label filtering with a stricter threshold and iterative refinement, and integration of large language models for pseudo-label generation and multimodal feature alignment—conducting more comprehensive domain generalization experiments covering cross-corpus, speaker-independent, and low-resource multilingual evaluations to validate real-world robustness, and extending the model to handle ambiguous and mixed emotions by modifying the loss function to support multi-label classification with soft labels for more nuanced emotion representation. These efforts will solidify the model’s standing as a prominent method in affective computing and broaden its suitability for a wider range of scenarios and domains.
